# Non-linear association between body weight and functional outcome after acute ischemic stroke

**DOI:** 10.1038/s41598-023-35894-y

**Published:** 2023-05-29

**Authors:** Kayo Wakisaka, Ryu Matsuo, Koutarou Matsumoto, Yasunobu Nohara, Fumi Irie, Yoshinobu Wakisaka, Tetsuro Ago, Naoki Nakashima, Masahiro Kamouchi, Takanari Kitazono

**Affiliations:** 1 Department of Medicine and Clinical Science, Graduate School of Medical Sciences, Kyushu University, Fukuoka, Japan; 2 Department of Health Care Administration and Management, Graduate School of Medical Sciences, Kyushu University, 3-1-1 Maidashi, Higashi-Ku, Fukuoka, 812-8582 Japan; 3 Biostatistics Center, Kurume University, Kurume, Japan; 4 Big Data Science and Technology, Faculty of Advanced Science and Technology, Kumamoto University, Kumamoto, Japan; 5 Medical Information Center, Kyushu University Hospital, Fukuoka, Japan; 6 Center for Cohort Studies, Graduate School of Medical Sciences, Kyushu University, Fukuoka, Japan

**Keywords:** Stroke, Risk factors, Weight management

## Abstract

This study aimed to determine whether body weight is associated with functional outcome after acute ischemic stroke. We measured the body mass index (BMI) and assessed clinical outcomes in patients with acute ischemic stroke. The BMI was categorized into underweight (< 18.5 kg/m^2^), normal weight (18.5–22.9 kg/m^2^), overweight (23.0–24.9 kg/m^2^), and obesity (≥ 25.0 kg/m^2^). The association between BMI and a poor functional outcome (modified Rankin Scale [mRS] score: 3–6) was evaluated. We included 11,749 patients with acute ischemic stroke (70.3 ± 12.2 years, 36.1% women). The risk of a 3-month poor functional outcome was higher for underweight, lower for overweight, and did not change for obesity in reference to a normal weight even after adjusting for covariates by logistic regression analysis. Restricted cubic splines and SHapley Additive exPlanation values in eXtreme Gradient Boosting model also showed non-linear relationships. Associations between BMI and a poor functional outcome were maintained even after excluding death (mRS score: 3–5) or including mild disability (mRS score: 2–6) as the outcome. The associations were strong in older patients, non-diabetic patients, and patients with mild stroke. Body weight has a non-linear relationship with the risk of a poor functional outcome after acute ischemic stroke.

## Introduction

The prevalence of obesity has continued to increase globally not only in high-income countries, but also in low or medium income countries^1–4^. Obesity is an established risk factor for a wide range of diseases, such as diabetes, cardiovascular diseases, sleep apnea, and cancer^5–8^. Therefore, controlling the global obesity epidemic is an important issue worldwide. However, a high body mass index (BMI) may paradoxically exert protective effects on mortality in the general population or in patients with several chronic diseases^9,10^, which is known as the “obesity paradox.”^11,12^.

Although a higher BMI is associated with a higher risk of ischemic stroke^13^, how BMI affects clinical outcomes after acute ischemic stroke is still unknown. Previous studies have reported inconsistent findings regarding the association between BMI and clinical outcomes in the short term after stroke^14–27^. Recent systematic reviews have pointed out several methodological issues in previous studies, such as insufficient consideration for multivariable adjustment, stroke subtypes, definitions of an abnormal body weight and clinical outcomes, and the follow-up period after onset^28–30^. In fact, a recent meta-analysis showed that the associations of BMI with post-stroke outcomes differed depending on whether these factors were taken into account^30^. As a result, whether the obesity paradox exists in the post-stroke prognosis remains controversial^31,32^.

The BMI reflects both fat mass and skeletal muscle mass. Fat mass is probably advantageous for recovery from severe illness, and skeletal muscle mass is crucial to improve the functional status after stroke^33–35^. Therefore, the obesity paradox may be applicable to patients with stroke as follows: as BMI increases in patients with stroke, a functional outcome and prognosis in the short term after onset might be better. A better knowledge of the relationship between BMI and post-stroke outcomes would lead to personalized care and rehabilitation in individual patients with acute stroke.

In this study, we aimed to determine whether overweight or obesity is beneficial regarding functional outcome and survival in the short term after acute ischemic stroke. We prospectively enrolled a large number of consecutive patients with acute ischemic stroke, measured their BMI on admission, and evaluated its association with the 3-month functional outcome and death after adjusting for potential confounding factors in a large-scale, multicenter, hospital-based stroke registry in Fukuoka, Japan. Furthermore, this relationship might not be simple but complicated instead. Therefore, we evaluated the associations not only by a conventional logistic regression model after categorization, but also by cubic spline logistic regression and SHapley Additive exPlanation (SHAP) values in a machine learning model.

## Results

### Baseline characteristics

Among 11,749 patients, the mean (standard deviation) age was 70.3 (12.2) years, and 36.1% were women. The baseline characteristics of the patients are shown according to the BMI categories in Table 1. Patients with a higher BMI were younger, and the proportion of male sex increased with an increase in BMI. The prevalence of hypertension, diabetes mellitus, and dyslipidemia was significantly higher, while atrial fibrillation and a pre-stroke modified Rankin Scale (mRS) score of 1 (no functional disability, despite symptoms) were less frequent with a higher BMI. Waist circumference increased as BMI increased. The proportion of small-vessel occlusion and large artery atherosclerosis increased, whereas that of cardioembolism decreased with an increase in BMI. Neurological symptoms were less severe and reperfusion therapy was less frequent with a high BMI.

**Table 1:** Baseline characteristics according to body mass index for the analysis of 3-month outcomes.

	Underweight (< 18.5 kg/m^2^) n = 916	Normal weight (18.5–22.9 kg/m^2^) n = 4840	Overweight (23.0–24.9 kg/m^2^) n = 2673	Obesity (≥ 25.0 kg/m^2^) n = 3320	*P*	*P*_trend_
Age, years, mean ± SD	76 ± 13	72 ± 12	70 ± 11	67 ± 12	< 0.001	< 0.001
Men, n (%)	415 (45.3)	2980 (61.6)	1882 (70.4)	2230 (67.2)	< 0.001	< 0.001
Risk factors						
Hypertension, n (%)	640 (69.9)	3641 (75.2)	2225 (83.2)	2902 (87.4)	< 0.001	< 0.001
Diabetes, n (%)	162 (17.7)	1327 (27.4)	819 (30.6)	1289 (38.8)	< 0.001	< 0.001
Dyslipidemia, n (%)	329 (35.9)	2480 (51.2)	1676 (62.7)	2273 (68.5)	< 0.001	< 0.001
Atrial fibrillation, n (%)	306 (33.4)	1132 (23.4)	521 (19.5)	593 (17.9)	< 0.001	< 0.001
Waist circumference, cm, mean ± SD	69.6 ± 6.3	79.3 ± 6.4	86.0 ± 5.8	94.2 ± 8.6	< 0.001	< 0.001
Previous stroke, n (%)	137 (15.0)	737 (15.2)	424 (15.9)	508 (15.3)	0.87	0.74
Pre-stroke modified Rankin scale score of 1, n (%)	175 (19.1)	624 (12.9)	284 (10.6)	344 (10.4)	< 0.001	< 0.001
Stroke subtype, n (%)						
Cardioembolism	284 (31.0)	1017 (21.0)	456 (17.1)	479 (14.4)	< 0.001	< 0.001
Small-vessel occlusion	215 (23.5)	1358 (28.1)	817 (30.6)	1094 (33.0)	< 0.001	< 0.001
Large artery atherosclerosis	107 (11.7)	777 (16.1)	428 (16.0)	568 (17.1)	0.001	0.003
Unclassified	310 (33.8)	1688 (34.9)	972 (36.4)	1179 (35.5)	0.45	0.27
Baseline NIHSS score, median (IQR)	4 (1–9)	2 (1–5)	2 (1–4)	2 (1–4)	< 0.001	< 0.001
Reperfusion therapy, n (%)	125 (13.6)	545 (11.3)	275 (10.3)	295 (8.9)	< 0.001	< 0.001

*P*_*trend*_ *P* for trend, *SD* standard deviation, *NIHSS* National Institutes of Health Stroke Scale, *IQR* interquartile range.

### Associations between BMI and clinical outcomes in the logistic regression model

We first evaluated the association between BMI and a poor functional outcome at 3 months after onset using a logistic regression analysis with categorized BMI groups (Table 2). We found that the odds ratios (ORs) of a poor functional outcome were significantly higher for underweight, lower for overweight, and remained comparable to obesity (vs. normal weight) after adjusting for potential confounding factors. Similar associations were found between BMI categories and the risk of functional disability or death from any cause (Table 2).

**Table 2:** Associations between BMI and unfavorable clinical outcomes at 3 months.

	Events, n (%)	Age- and sex-adjusted			Multivariable-adjusted		
		OR	(95% CI)	*P*	OR	(95% CI)	*P*
Poor functional outcome							
Underweight (11.8–18.4 kg/m^2^), n = 916	375 (40.9)	1.75	(1.50–2.05)	< 0.001	1.52	(1.24–1.85)	< 0.001
Normal weight (18.5–22.9 kg/m^2^), n = 4840	1158 (23.9)	1.00	Reference		1.00	Reference	
Overweight (23.0–24.9 kg/m^2^), n = 2673	473 (17.7)	0.79	(0.69–0.89)	< 0.001	0.83	(0.71–0.96)	0.02
Obesity (25.0–60.5 kg/m^2^), n = 3320	578 (17.4)	0.89	(0.80–1.00)	0.06	0.97	(0.81–1.15)	0.71
Functional disability							
Underweight (11.8–18.4 kg/m^2^), n = 857	316 (36.9)	1.63	(1.38–1.91)	< 0.001	1.41	(1.15–1.74)	0.001
Normal weight (18.5–22.9 kg/m^2^), n = 4728	1046 (22.1)	1.00	Reference		1.00	Reference	
Overweight (23.0–24.9 kg/m^2^), n = 2641	441 (16.7)	0.81	(0.72–0.92)	0.002	0.86	(0.73–1.00)	0.06
Obesity (25.0–60.5 kg/m^2^), n = 3285	543 (16.5)	0.93	(0.82–1.05)	0.23	0.98	(0.82–1.18)	0.86
Death							
Underweight (11.8–18.4 kg/m^2^), n = 916	59 (6.4)	2.47	(1.78–3.45)	< 0.001	1.96	(1.32–2.93)	0.001
Normal weight (18.5–22.9 kg/m^2^), n = 4840	112 (2.3)	1.00	Reference		1.00	Reference	
Overweight (23.0–24.9 kg/m^2^), n = 2673	32 (1.2)	0.56	(0.38–0.84)	0.005	0.59	(0.38–0.91)	0.02
Obesity (25.0–60.5 kg/m^2^), n = 3320	35 (1.1)	0.57	(0.39–0.85)	0.005	0.63	(0.38–1.03)	0.06

ORs and 95% CIs of each outcome of interest were estimated for underweight, overweight, and obesity vs. normal weight as a reference. The multivariable model included age, sex, hypertension, diabetes mellitus, dyslipidemia, atrial fibrillation, waist circumference, prior stroke, pre-stroke functional status, stroke subtype, the National Institutes of Health Stroke Scale score on admission, and reperfusion therapy.

*BMI* body mass index, *OR* odds ratio, *CI* confidence interval.

**Figure 1:**
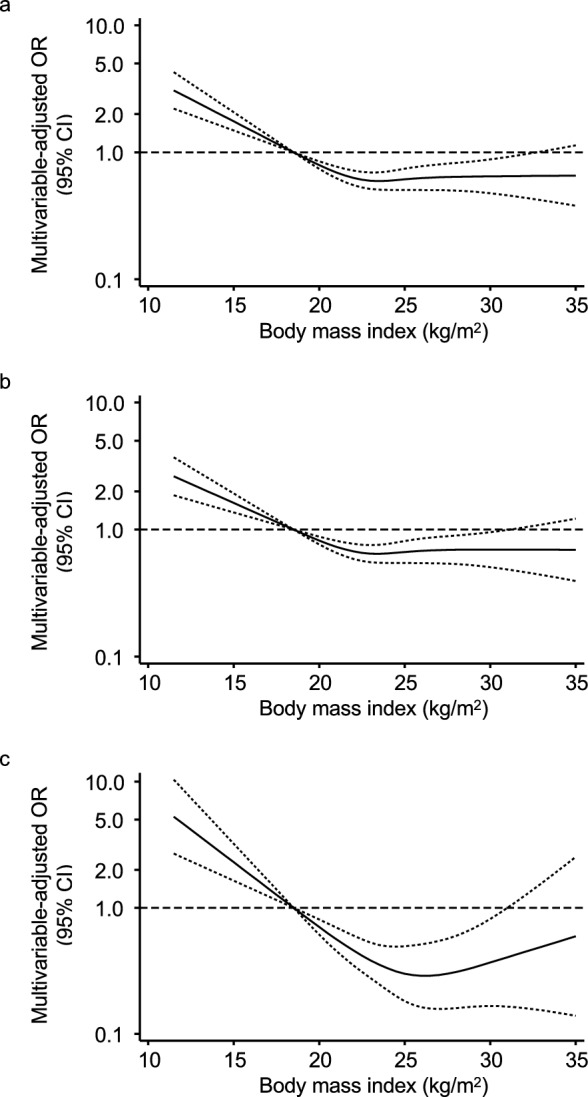
Relationship between body mass index and unfavorable clinical outcomes at 3 months. The associations between body mass index and the risk of a poor functional outcome (**a**), functional disability (**b**), or death (**c**) at 3 months after onset are shown. ORs (solid lines) and 95% CIs (dotted lines) were estimated in reference to a body mass index of 18.5 kg/m^2^ using a logistic regression model and are shown by restricted cubic spline curves. The multivariable model included age, sex, hypertension, diabetes mellitus, dyslipidemia, atrial fibrillation, waist circumference, prior stroke, pre-stroke functional status, stroke subtype, the National Institutes of Health Stroke Scale score on admission, and reperfusion therapy. *OR* odds ratio, *CI* confidence interval.

We further confirmed the non-linear relationship between BMI and 3-month unfavorable outcomes by a restricted cubic spline curve. We found that the estimated ORs of a poor functional outcome at 3 months were increased for underweight, decreased for normal weight and overweight, and did not change for obesity in reference to a BMI of 18.5 kg/m^2^ (Fig. 1a). Similar relationships were also observed for the risk of functional disability (Fig. 1b) and all-cause death (Fig. 1c).

### Associations between BMI and clinical outcomes in the decision tree ensemble model

**Figure 2:**
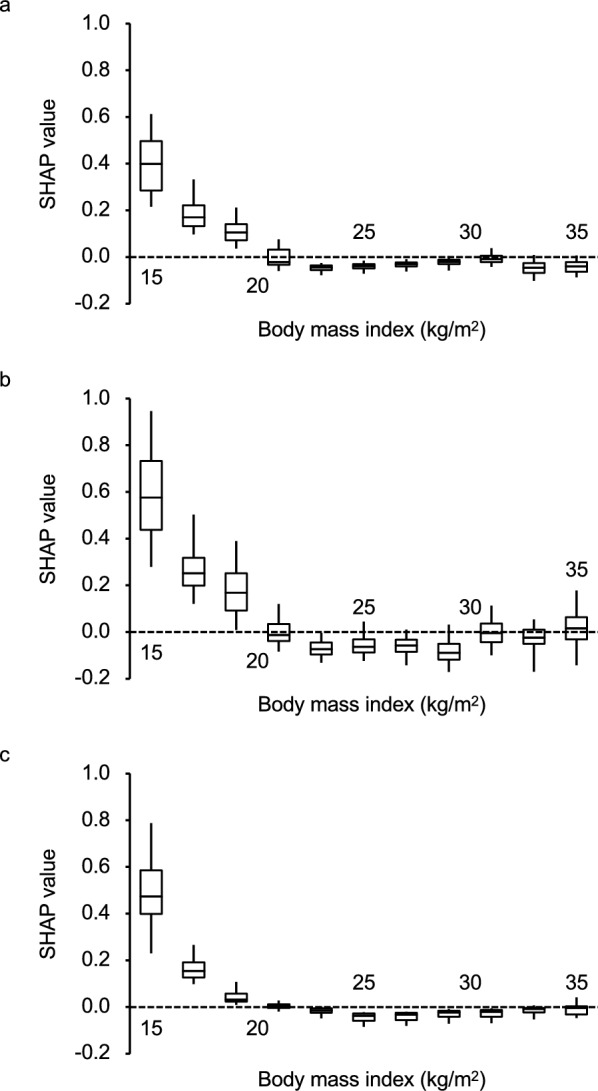
SHAP values of body mass index for unfavorable clinical outcomes at 3 months. SHAP values of the body mass index in predicting a poor functional outcome (**a**), functional disability (**b**), or death (**c**) at 3 months after onset in 11 body mass index categories are shown. SHAP values for post-stroke clinical outcomes were calculated in a decision tree ensemble model. Boxes, horizontal lines, and vertical lines indicate the interquartile range, median, and 5–95 percentiles, respectively, of SHAP values for each clinical outcome in 11 groups according to body mass index in increments of 2 (body mass index groups: < 16 kg/m^2^, increment of 2 from 16 to 33.9 kg/m^2^, and ≥ 34 kg/m^2^). *SHAP* SHapley Additive exPlanation.

The shape of the cubic spline curve can be altered depending on the reference value of BMI. Therefore, we determined the relationship between a wide range of BMIs and SHAP values for a 3-month poor functional outcome in individual patients. In the decision tree ensemble model, SHAP values of BMI were high for underweight, but low for normal weight, overweight, and obesity in predicting a poor functional outcome at 3 months (see Supplementary Table S1 online). These associations were maintained for functional disability and all-cause death. SHAP values showed similar non-linear relationships for the risks of a poor functional outcome (Fig. 2a), functional disability (Fig. 2b), and all-cause death (Fig. 2c).

### Subgroup analysis

We then performed subgroup analyses to determine whether there are specific populations whose functional outcomes are susceptible to BMI. We investigated effect modification of the association between BMI and a 3-month poor functional outcome for age, sex, risk factors (hypertension, diabetes mellitus, dyslipidemia, and atrial fibrillation), stroke subtype, stroke severity, previous stroke, and reperfusion therapy.

Heterogeneity was found according to age, diabetes mellitus, and initial stroke severity, although no interaction was found according to the other factors (*P* for heterogeneity: 0.07–0.86). This relationship appeared to be strong in patients aged ≥ 65 years, non-diabetic patients, and patients with a National Institutes of Health Stroke Scale (NIHSS) score ≤ 6 (see Supplementary Fig. S1 online).

Plots of SHAP values versus BMI showed that the relationships appeared to be altered by age and stroke severity, but the change in the relationship appeared to be small according to diabetes mellitus (see Supplementary Fig. S2 online).

### Sensitivity analysis

We performed sensitivity analyses to determine whether the associations were still robust even when clinical outcomes were evaluated at an earlier timing. In a cohort of 11,989 patients (70.3 ± 12.2 years, women: 36.1%, see Supplementary Table S2 online), similar trends for discharge outcomes were maintained between BMI and the risk of unfavorable clinical outcomes (Table 3, see Supplementary Figs. S3 and S4 online). These associations were less remarkable compared with those at 3 months in the main cohort.

**Table 3:** Associations between BMI and unfavorable clinical outcomes at discharge.

	Events, n (%)	Age- and sex-adjusted			Multivariable-adjusted		
		OR	(95% CI)	*P*	OR	(95% CI)	*P*
Poor functional outcome							
Underweight (11.8–18.4 kg/m^2^), n = 939	394 (42.0)	1.53	(1.32–1.78)	< 0.001	1.25	(1.03–1.53)	0.02
Normal weight (18.5–22.9 kg/m^2^), n = 4934	1382 (28.0)	1.00	Reference		1.00	Reference	
Overweight (23.0–24.9 kg/m^2^), n = 2730	584 (21.4)	0.78	(0.69–0.87)	< 0.001	0.80	(0.70–0.93)	0.003
Obesity (25.0–60.5 kg/m^2^), n = 3386	719 (21.2)	0.88	(0.79–0.98)	0.02	0.92	(0.78–1.08)	0.32
Functional disability							
Underweight (11.8–18.4 kg/m^2^), n = 910	365 (40.1)	1.48	(1.27–1.72)	< 0.001	1.22	(1.00–1.49)	0.048
Normal weight (18.5–22.9 kg/m^2^), n = 4881	1329 (27.2)	1.00	Reference		1.00	Reference	
Overweight (23.0–24.9 kg/m^2^), n = 2712	566 (20.9)	0.78	(0.70–0.88)	< 0.001	0.81	(0.70–0.94)	0.005
Obesity (25.0–60.5 kg/m^2^), n = 3370	703 (20.9)	0.89	(0.80–0.99)	0.03	0.92	(0.78–1.09)	0.34
Death							
Underweight (11.8–18.4 kg/m^2^), n = 939	29 (3.1)	2.43	(1.52–3.88)	< 0.001	1.92	(1.08–3.39)	0.03
Normal weight (18.5–22.9 kg/m^2^), n = 4934	53 (1.1)	1.00	Reference		1.00	Reference	
Overweight (23.0–24.9 kg/m^2^), n = 2730	18 (0.7)	0.69	(0.40–1.18)	0.17	0.72	(0.39–1.30)	0.27
Obesity (25.0–60.5 kg/m^2^), n = 3386	16 (0.5)	0.58	(0.33–1.03)	0.06	0.65	(0.32–1.35)	0.25

ORs and 95% CIs of each outcome of interest were estimated for underweight, overweight, and obesity vs. normal weight as a reference. The multivariable model included age, sex, hypertension, diabetes mellitus, dyslipidemia, atrial fibrillation, waist circumference, prior stroke, pre-stroke functional status, stroke subtype, the National Institutes of Health Stroke Scale score on admission, and reperfusion therapy.

*BMI* body mass index, *OR* odds ratio, *CI* confidence interval.

We set the cut-off point of the mRS score for unfavorable outcomes to a milder level (i.e., mRS score: ≥ 2). Nevertheless, the associations were essentially unchanged (Table 4).

**Table 4:** Associations between BMI and mild functional disability at 3 months.

	Events, n (%)	Age- and sex-adjusted			Multivariable-adjusted		
		OR	(95% CI)	*P*	OR	(95% CI)	*P*
Poor functional outcome							
Underweight (11.8–18.4 kg/m^2^), n = 916	493 (53.8)	1.68	(1.45–1.95)	< 0.001	1.43	(1.19–1.72)	< 0.001
Normal weight (18.5–22.9 kg/m^2^), n = 4840	1772 (36.6)	1.00	Reference		1.00	Reference	
Overweight (23.0–24.9 kg/m^2^), n = 2673	751 (28.1)	0.75	(0.67–0.83)	< 0.001	0.78	(0.69–0.89)	< 0.001
Obesity (25.0–60.5 kg/m^2^), n = 3320	951 (28.6)	0.87	(0.79–0.96)	0.007	0.93	(0.80–1.08)	0.33
Functional disability							
Underweight (11.8–18.4 kg/m^2^), n = 857	434 (50.6)	1.58	(1.36–1.84)	< 0.001	1.36	(1.13–1.64)	0.001
Normal weight (18.5–22.9 kg/m^2^), n = 4728	1660 (35.1)	1.00	Reference		1.00	Reference	
Overweight (23.0–24.9 kg/m^2^), n = 2641	719 (27.2)	0.76	(0.69–0.85)	< 0.001	0.80	(0.70–0.91)	0.001
Obesity (25.0–60.5 kg/m^2^), n = 3285	916 (27.9)	0.89	(0.81–0.99)	0.03	0.94	(0.80–1.09)	0.39

ORs and 95% CIs of each outcome of interest were estimated for underweight, overweight, and obesity versus normal weight as a reference. The multivariable model included age, sex, hypertension, diabetes mellitus, dyslipidemia, atrial fibrillation, waist circumference, prior stroke, pre-stroke functional status, stroke subtype, the National Institutes of Health Stroke Scale score on admission, and reperfusion therapy.

*BMI* body mass index, *OR* odds ratio, *CI* confidence interval, *mRS* modified Rankin Scale.

Additionally, we confirmed the associations using the criteria of the World Health Organization (WHO) in which normal weight and overweight were grouped into normal weight (see Supplementary Table S3 online). We found that multivariable-adjusted ORs of a poor functional outcome at 3 months were increased for underweight, did not change for overweight, and were increased for obesity in reference to normal weight (see Supplementary Table S4 online).

## Discussion

The major findings in this study were as follows. The logistic regression model and decision tree ensemble model showed that BMI had a unique non-linear relationship with the functional outcome after ischemic stroke as follows: the risk of a poor functional outcome was increased for underweight and decreased for overweight, but was not further decreased for obesity in reference to normal weight. Similar associations were found for risks of functional disability in survivors and all-cause death at 3 months. These associations were maintained at an earlier timing and for mild functional disability. There was heterogeneity in the association of BMI with the functional outcome according to some patients’ characteristics. The present study sheds new light on body weight as a significant predictor that has a complex relationship with clinical outcomes after acute ischemic stroke.

Previous studies reported inconsistent findings on the associations between BMI and functional outcomes after stroke. Some studies reported that overweight or obesity was not associated with functional outcomes after ischemic stroke^18,19,21,23^, whereas a favorable association with 3-month functional outcomes was reported in other studies in patients with ischemic^17,22^ or hemorrhagic stroke^20^. Some studies showed a significant association of underweight with a poor functional outcome after ischemic stroke^18,19^; however, this association was not reported in other studies for 3-month outcomes after ischemic stroke^17,19,23^. Moreover, a bell-shaped relationship between a high or low BMI and worse outcomes was shown in patients with successful reperfusion after mechanical thrombectomy^24^.

In the present cohort, the risk of unfavorable functional outcomes was greatly increased for underweight and decreased for overweight, but did not further change for obesity according to the Western Pacific Region of the World Health Organization (WPRO) criteria. Moreover, the risk of unfavorable functional outcomes tended to be increased for obesity when the WHO criteria were applied. These findings clearly indicate that underweight is disadvantageous for functional outcomes, and that beneficial effects of BMI on the post-stroke functional outcome only occur for overweight, but not for obesity. Our study suggests that the relationship between BMI and post-stroke functional outcomes is not linear but complex. This relationship is L-shaped when using the WPRO criteria for Asians and U-shaped when using the WHO criteria. Our findings do not suggest that a higher BMI is associated with a better post-stroke functional status.

Whether the obesity paradox is present for short-term death after stroke is controversial. Some studies showed a beneficial association of overweight or obesity with survival after stroke^22,25,26,36^, which supports the presence of an obesity paradox. However, other studies showed no association^14–16,23,27^ or a detrimental association^17^ of overweight or obesity with the risk of death after stroke. Regarding underweight, some studies are in agreement with its disadvantageous effects on death after stroke^15,16,27^. However, other studies reported no association of underweight with the risk of death after intracerebral hemorrhage^14^ or after mechanical thrombectomy^23^. Our study showed that the short-term risk of all-cause death was greatly increased for underweight, but decreased for overweight, and slightly increased with an increase in BMI for obesity. Consequently, underweight patients have a higher risk of post-stroke death than normal weight patients, and patients with obesity do not have an advantage for the short-term risk of death compared with normal weight patients. Therefore, in the present cohort, the obesity paradox was not obvious in short-term death after ischemic stroke.

Although many studies have investigated the relationship between body weight and post-stroke outcomes, the findings have varied. The reasons for the differences between studies may be attributed to the definitions of overweight and obesity and/or the reference group (normal weight or other grouping) because the relationship is not linear but L- or U-shaped. Additionally, the discrepancies between studies may be caused by differences in the definitions of outcomes (functional disability only in survivors, poor functional outcome, or death), the multivariable model used, evaluation timing of outcome (short term [e.g., discharge, 30 days, or 90 days] or long term), or racial and ethnic groups. Recent systematic reviews have concluded that higher evidence is still required to clarify the relationship between obesity and stroke outcomes because there were several methodological concerns in previous studies^28,29^. To address these methodological issues, we prospectively measured body weight on admission in consecutive patients to avoid inaccuracy of BMI and to exclude any selection bias. We adjusted for comorbid conditions and stroke characteristics to control for potential confounding factors. We also assessed the clinical outcomes at the same timing after onset to standardize the timing of evaluation. Furthermore, we analyzed the non-linear relationship by the decision tree ensemble model to avoid the effect of variables with a non-linear relationship and interactions, as well as the reference value of BMI. Further studies using adequate methods are required to determine the association of BMI with clinical outcomes after stroke in other settings.

There may be some plausible explanations for the reasons why body weight has such a complicated relationship with post-stroke clinical outcomes (i.e., an unfavorable relationship for underweight and a favorable relationship for overweight, but not obesity). First, adiposity may serve as a reserve against metabolic and energy imbalance after stroke. Stroke induces various acute pathophysiological responses, most of which lead to increased catabolic drive and suppressed anabolic stimulation^37^. This imbalance together with inactivity and paresis accelerate weight loss and lead to poor outcomes after stroke, whereas fat mass may be protective against this malignant cycle. Second, because skeletal muscle mass is an independent predictor for post-stroke functional outcomes, BMI may reflect the beneficial effects of skeletal muscle on the functional outcome^34,35^. Third, adipose tissue and/or skeletal muscle might function as endocrine systems to mitigate ischemic damage in the brain or facilitate recovery from ischemic stroke. Even if these possibilities are true, questions arise as to why the beneficial association of BMI with the risk of unfavorable outcomes disappears or reverses with an increase in BMI in patients with obesity. Some possible mechanisms may underlie this non-linear relationship. One possible explanation is that enlarged adipose tissue mass can cause insulin resistance^38^, which potentially counteracts the beneficial effects of adipose tissue^39^. Another possibility is that a heavy weight may hamper functional recovery or simply interfere with daily activities because of weight itself or accompanying orthopedic problems.

In our subgroup analyses, the unique association between BMI and the 3-month functional outcome appeared to be remarkable in older patients, non-diabetic patients, and patients with non-severe neurological symptoms. In these patients, more attention is required for body weight regarding post-stroke clinical outcomes. Future studies are needed to determine the mechanisms of the unique relationship between body weight and the risks of unfavorable functional outcomes after ischemic stroke.

This study has some limitations that should be considered. First, patients whose data were lacking or patients who were lost during follow-up were excluded from the analysis, which led to selection bias. Second, we used the WPRO criteria to categorize BMI groups in Japanese patients, although similar results were also obtained using the WHO criteria. Third, this was an observational study. Therefore, the causal relationship between body weight and clinical outcomes cannot be determined. Fourth, because the follow-up time was short, the potential adverse effects of obesity on the stroke prognosis may have been underestimated. Finally, the present study was performed in a restricted region in Japan. Generalizability needs to be examined in other studies with different race–ethnic groups.

In conclusion, body weight has a non-linear relationship not only with the prognosis, but also with the functional outcome, in the short term after onset. Underweight appears to have an unfavorable relationship, whereas overweight, but not obesity, is relatively favorable. Further study is warranted to determine the clinical implications of BMI in clinical practice in the management of patients with stroke.

## Methods

### Study design and setting

We constructed the Fukuoka Stroke Registry, which is a multicenter, hospital-based registry of Japanese patients with acute stroke who were hospitalized in seven participating stroke centers in Fukuoka, Japan (UMIN Clinical Trial Registry: UMIN-CTR, Unique ID: UMIN000000800, 2007/9/1). The study design was approved by the Institutional Review Boards of all of the following participating hospitals: Kyushu University Institutional Review Board for Clinical Research, 22086-00; Kyushu Medical Center Institutional Review Board, R06-03; Clinical Research Review Board of Fukuokahigashi Medical Center, 29-C-38; Fukuoka Red Cross Hospital Institutional Review Board, 629; St. Mary’s Hospital Research Ethics Review Committee, S13-0110; Steel Memorial Yawata Hospital Ethics Committee, 06-04-13; and Kyushu Rosai Hospital Institutional Review Board, 21-8. The Fukuoka Stroke Registry enrolled consecutive patients with acute stroke within 7 days after onset. We obtained written informed consent from all patients or their family members for the prospective study. All methods were performed in accordance with the relevant guidelines and regulations. Details of the Fukuoka Stroke Registry have been described elsewhere^40,41^.

### Study patients

Stroke was defined as the sudden onset of nonconvulsive and focal neurological deficits. Ischemic stroke was diagnosed by computed tomography, magnetic resonance imaging, or both. From June 2007 to September 2019, 15,569 patients with acute ischemic stroke were initially enrolled for the prospective study in the Fukuoka Stroke Registry. Of them, we excluded 3386 patients who were functionally dependent before admission (mRS score ≥ 2) to avoid the influence of functional status before stroke onset. We further excluded 194 patients whose data on variables for multivariable analysis were lacking. Additionally, we excluded 240 patients who were lost during the follow-up period of 3 months. Finally, we included 11,749 patients in the main analysis for 3-month outcomes. In a sensitivity analysis for discharge outcomes, all 11,989 patients who were discharged from the hospitals were included in the analysis. A flow chart of selecting the patients is shown in Supplementary Fig. S5.

### BMI

Body weight was measured on admission for index stroke. We calculated BMI by dividing body weight in kilograms by height in meters squared. The BMI was categorized into four groups in accordance with the criteria for Asians proposed by the Regional Office for the WPRO as follows: underweight (< 18.5 kg/m^2^), normal weight (18.5 to < 23 kg/m^2^), overweight (23 to < 25 kg/m^2^), and obesity (≥ 25 kg/m^2^)^42^. In the sensitivity analysis, we categorized BMI using the criteria of the WHO as underweight (< 18.5 kg/m^2^), normal weight (18.5 to < 25 kg/m^2^), overweight (25 to < 29.9 kg/m^2^), and obesity (≥ 30 kg/m^2^)^43^.

### Clinical variables

We assessed baseline clinical variables to adjust for potential confounders in the logistic regression model. They included demographics (age and sex), risk factors (hypertension, diabetes mellitus, dyslipidemia, and atrial fibrillation), waist circumference, previous stroke, pre-stroke functional status, stroke subtypes, neurological severity (NIHSS score), and reperfusion therapy during hospitalization of index stroke. The definitions of these covariates are shown in the Supplemental Methods. Other variables were also used in developing the machine learning model (Supplemental Methods).

### Study outcomes

The main study outcome was a poor functional outcome (mRS score: 3–6) at 3 months after onset. Because the main outcome included death after stroke onset, we separately evaluated functional disability in survivors (mRS score: 3–5) at 3 months and death from any cause (mRS score: 6) within 3 months after onset as the secondary outcomes. In the sensitivity analyses, these clinical outcomes were evaluated at discharge or by including mild functional disability (mRS score: 2) in an unfavorable functional outcome.

### Statistical analysis

We evaluated the difference in baseline characteristics among BMI categories using the chi-square test or Kruskal–Wallis test. Trends in baseline characteristics according to BMI groups were assessed by the Jonckheere–Terpstra test for continuous variables and the Cochran–Armitage trend test for category variables. We performed a logistic regression analysis to adjust for covariates, and estimated ORs and 95% confidence intervals of each outcome of interest. The non-linear relationship between BMI and post-stroke clinical outcomes was confirmed by a restricted cubic spline. The multivariable model included age, sex, risk factors (hypertension, diabetes mellitus, dyslipidemia, and atrial fibrillation), waist circumference, a previous history of stroke, pre-stroke functional status (mRS score of 0 or 1 before onset), stroke subtype (cardioembolism, small-vessel occlusion, large artery atherosclerosis, or others), the NIHSS score on admission, and reperfusion therapy.

To further evaluate the contribution of BMI to post-stroke clinical outcomes, we developed a decision tree ensemble model with eXtreme Gradient Boosting using all baseline variables. In this model, we implemented SHAP to estimate the contribution of BMI in predicting the outcomes^44^. The SHAP value represents the contribution of the weight of each variable to the prediction model, which is considered comparable to a standardized partial regression coefficient in linear regression models^45^.

A subgroup analysis was performed in the groups stratified by age (< 65 or ≥ 65 years), sex, risk factors (hypertension, diabetes mellitus, dyslipidemia, and atrial fibrillation), stroke subtype (cardioembolism or non-cardioembolism), stroke severity (NIHSS score on admission ≤ 6 or > 6), previous stroke, or reperfusion therapy. Heterogeneity was evaluated by the likelihood ratio test after adding an interaction term of the BMI categories × subgroup.

Two-tailed *P* < 0.05 was considered statistically significant. Statistical analyses were performed using STATA version 16.0 (StataCorp LLC, College Station, TX, USA) and R statistical package (http://www.r-project.org/, version 4.1.0).

## Supplementary Information

Supplementary Information.

## Data Availability

The datasets generated during and/or analyzed during the current study are not publicly available owing to the sensitive nature of the data collected for this study, but are available from the corresponding author on reasonable request.

## References

[1] Worldwide trends in body-mass index (2017). underweight, overweight, and obesity from 1975 to 2016: A pooled analysis of 2416 population-based measurement studies in 128·9 million children, adolescents, and adults. Lancet.

[2] Afshin A (2017). Health effects of overweight and obesity in 195 countries over 25 years. N. Engl. J. Med..

[3] Bluher M (2019). Obesity: Global epidemiology and pathogenesis. Nat. Rev. Endocrinol..

[4] Malik VS, Willet WC, Hu FB (2020). Nearly a decade on—Trends, risk factors and policy implications in global obesity. Nat. Rev. Endocrinol..

[5] Field AE (2001). Impact of overweight on the risk of developing common chronic diseases during a 10-year period. Arch. Intern. Med..

[6] Whitlock G, Lewington S, Mhurchu CN (2002). Coronary heart disease and body mass index: A systematic review of the evidence from larger prospective cohort studies. Semin. Vasc. Med..

[7] Young T, Peppard PE, Gottlieb DJ (2002). Epidemiology of obstructive sleep apnea: A population health perspective. Am. J. Respir. Crit. Care Med..

[8] Lauby-Secretan B (2016). Body fatness and cancer–viewpoint of the IARC Working Group. N. Engl. J. Med..

[9] Oreopoulos A (2008). Body mass index and mortality in heart failure: A meta-analysis. Am. Heart J..

[10] Druml W (2020). Association of body mass index and outcome in acutely ill patients with chronic kidney disease requiring intensive care therapy. J. Ren. Nutr..

[11] Romero-Corral A (2006). Association of bodyweight with total mortality and with cardiovascular events in coronary artery disease: A systematic review of cohort studies. Lancet.

[12] Lavie CJ, Milani RV, Ventura HO (2009). Obesity and cardiovascular disease: Risk factor, paradox, and impact of weight loss. J. Am. Coll. Cardiol..

[13] Strazzullo P (2010). Excess body weight and incidence of stroke: Meta-analysis of prospective studies with 2 million participants. Stroke.

[14] Kim BJ (2011). Paradoxical longevity in obese patients with intracerebral hemorrhage. Neurology.

[15] Kim BJ (2012). Dynamics of obesity paradox after stroke, related to time from onset, age, and causes of death. Neurology.

[16] Dehlendorff C, Andersen KK, Olsen TS (2014). Body mass index and death by stroke: No obesity paradox. JAMA Neurol..

[17] Zhao L (2014). Favorable functional recovery in overweight ischemic stroke survivors: Findings from the China National Stroke Registry. J. Stroke Cerebrovasc. Dis..

[18] Kawase S (2017). Association between body mass index and outcome in Japanese ischemic stroke patients. Geriatr. Gerontol. Int..

[19] Sun W (2017). Association of body mass index with mortality and functional outcome after acute ischemic stroke. Sci. Rep..

[20] Dangayach NS (2018). Does the obesity paradox predict functional outcome in intracerebral hemorrhage?. J. Neurosurg..

[21] Chen W (2019). Association of body mass index and risk of stroke after acute minor stroke or TIA: A post hoc analysis of a randomized controlled trial. Neurotox. Res..

[22] Pirson FAV (2019). The effect of body mass index on outcome after endovascular treatment in acute ischemic stroke patients: A post hoc analysis of the MR CLEAN Trial. Cerebrovasc. Dis..

[23] Bouslama M (2020). Body mass index and clinical outcomes in large vessel occlusion acute ischemic stroke after endovascular therapy. Interv. Neurol..

[24] Chen SH (2020). Effect of body mass index on outcomes of mechanical thrombectomy in acute ischemic stroke. World Neurosurg..

[25] Javalkar V, Kuybu O, Davis D, Kelley RE (2020). Factors associated with inpatient mortality after intracerebral hemorrhage: Updated information from the United States Nationwide Inpatient Sample. J. Stroke Cerebrovasc. Dis..

[26] Chaudhary D (2021). Obesity and mortality after the first ischemic stroke: Is obesity paradox real?. PLoS ONE.

[27] Cao Z (2021). Body mass index and clinical outcomes in patients with intracerebral haemorrhage: Results from the China Stroke Center Alliance. Stroke Vasc. Neurol..

[28] Oesch L, Tatlisumak T, Arnold M, Sarikaya H (2017). Obesity paradox in stroke—Myth or reality? A systematic review. PLoS ONE.

[29] Forlivesi S, Cappellari M, Bonetti B (2021). Obesity paradox and stroke: A narrative review. Eat. Weight Disord..

[30] Zhang P, Yan XL, Qu Y, Guo ZN, Yang Y (2021). Association between abnormal body weight and stroke outcome: A meta-analysis and systematic review. Eur. J. Neurol..

[31] Bagheri M, Speakman JR, Shabbidar S, Kazemi F, Djafarian K (2015). A dose-response meta-analysis of the impact of body mass index on stroke and all-cause mortality in stroke patients: A paradox within a paradox. Obes. Rev..

[32] Huang K (2016). Association of BMI with total mortality and recurrent stroke among stroke patients: A meta-analysis of cohort studies. Atherosclerosis.

[33] Doehner W, Clark A, Anker SD (2010). The obesity paradox: Weighing the benefit. Eur. Heart J..

[34] Abe T (2020). Low muscle mass is associated with walking function in patients with acute ischemic stroke. J. Stroke Cerebrovasc. Dis..

[35] Ohyama K (2020). Correlation between skeletal muscle mass deficit and poor functional outcome in patients with acute ischemic stroke. J. Stroke Cerebrovasc. Dis..

[36] Hoffman H, Jalal MS, Furst T, Chin LS (2020). The obesity paradox in spontaneous intracerebral hemorrhage: Results from a retrospective analysis of the nationwide inpatient sample. Neurocrit. Care.

[37] Scherbakov N, Dirnagl U, Doehner W (2011). Body weight after stroke: Lessons from the obesity paradox. Stroke.

[38] Després JP, Lemieux I (2006). Abdominal obesity and metabolic syndrome. Nature.

[39] Ago T (2018). Insulin resistance and clinical outcomes after acute ischemic stroke. Neurology.

[40] Kamouchi M (2011). Prestroke glycemic control is associated with the functional outcome in acute ischemic stroke: The Fukuoka Stroke Registry. Stroke.

[41] Kumai Y (2012). Proteinuria and clinical outcomes after ischemic stroke. Neurology.

[42] Anuurad E (2003). The new BMI criteria for Asians by the regional office for the western pacific region of WHO are suitable for screening of overweight to prevent metabolic syndrome in elder Japanese workers. J. Occup. Health.

[43] WHO Consultation on Obesity & World Health Organization (2000). Obesity : Preventing and Managing the Global Epidemic: Report of a WHO Consultation (WHO technical report series) 1–252.

[44] Lundberg SM (2020). From local explanations to global understanding with explainable AI for trees. Nat. Mach. Intell..

[45] Inoguchi T, Nohara Y, Nojiri C, Nakashima N (2021). Association of serum bilirubin levels with risk of cancer development and total death. Sci. Rep..

