# Supplementary material for: Non-linear association between body weight and functional outcome after acute ischemic stroke
Source: Sci Rep. 2023 May 29;13:8697. doi: 10.1038/s41598-023-35894-y (PMC10226986; doi:10.1038/s41598-023-35894-y)
Supplement: Supplementary file 1 — Supplementary Information. [file 41598_2023_35894_MOESM1_ESM.docx]

**Supplementary Information**

**Non-linear association between body weight and functional outcome after acute ischemic stroke**

Kayo Wakisaka, MD, Ryu Matsuo, MD, PhD, Koutarou Matsumoto, MPH, PhD, Yasunobu Nohara, PhD, Fumi Irie, MD, PhD, Yoshinobu Wakisaka, MD, PhD, Tetsuro Ago, MD, PhD, Naoki Nakashima, MD, PhD, Masahiro Kamouchi, MD, PhD, Takanari Kitazono, MD, PhD

**Methods**

**References**

**Tables**

Supplementary Table S1. SHAP values of BMI for unfavorable clinical outcomes at 3 months.

Supplementary Table S2. Baseline characteristics according to BMI for analysis of discharge outcomes.

Supplementary Table S3. Baseline characteristics according to BMI based on World Health Organization criteria for analysis of 3-month outcomes.

Supplementary Table S4. Associations between BMI based on World Health Organization criteria and unfavorable clinical outcomes at 3 months

**Figures**

Supplementary Figure S1. Association between body mass index and a poor functional outcome at 3 months according to age, diabetes mellitus, and neurological severity.

Supplementary Figure S2. SHAP values of body mass index for a poor functional outcome at 3 months according to age, diabetes mellitus, and neurological severity.

Supplementary Figure S3. Relationships between body mass index and unfavorable clinical outcomes at discharge.

Supplementary Figure S4. SHAP values of body mass index for unfavorable clinical outcomes at discharge.

Supplementary Figure S5. Flow chart of the patients.

**Appendix**

**Methods**

***Fukuoka Stroke Registry***

Participating hospitals of the Fukuoka Stroke Registry were Kyushu University Hospital (Fukuoka, Japan), National Hospital Organization Kyushu Medical Center (Fukuoka, Japan), National Hospital Organization Fukuoka-Higashi Medical Center (Koga, Japan), Fukuoka Red Cross Hospital (Fukuoka, Japan), St Mary’s Hospital (Kurume, Japan), Steel Memorial Yawata Hospital (Kitakyushu, Japan), and Japan Labor Health and Welfare Organization Kyushu Rosai Hospital (Kitakyushu, Japan).

***Clinical assessments***

Baseline clinical data and the discharge outcome were evaluated by stroke neurologists and reviewed by members of the study steering committee. A follow-up study was performed by well-trained authorized nurses using a standardized questionnaire, in person, or by telephone at 3 months. Clinical outcomes were adjudicated by members of the event adjudication committee at 3 months.

The definitions of risk factors were as follows: hypertension (systolic blood pressure ≥140 mmHg, diastolic blood pressure ≥90 mmHg in the chronic stage [approximately 3 weeks after stroke onset], or a history of antihypertensive medication), diabetes mellitus (according to the diagnostic criteria of the Japan Diabetes Society^1^ in the chronic stage or a history of antidiabetic treatment), dyslipidemia (low-density lipoprotein cholesterol concentrations ≥3.62 mmol/L, high-density lipoprotein cholesterol concentrations <1.03 mmol/L, or triglyceride concentrations ≥1.69 mmol/L in the chronic stage, or a history of lipid-lowering medication), and atrial fibrillation (based on electrocardiographic findings on admission or during hospitalization, or a history of paroxysmal atrial fibrillation). The pre-stroke functional status was evaluated by the modified Rankin Scale (mRS) score. A pre-stroke mRS score of 1 was regarded as having symptoms without considerable disability among all eligible patients who were able to carry out all usual duties and activities before stroke onset (mRS score: 0–1) after excluding patients who were functionally dependent before stroke onset (mRS score: ≥2). Waist circumference was measured on admission of index stroke. A previous stroke included a history of hemorrhagic or ischemic stroke. Ischemic stroke subtypes were classified into four subtypes on the basis of the criteria of the Trial of Org 10172 in Acute Stroke Treatment study with miner modification.^2^ Neurological severity on admission was evaluated by the National Institutes of Health Stroke Scale (NIHSS). Reperfusion therapy included the use of thrombolytic agents, such as the recombinant tissue-type, mechanical thrombectomy, or both.

***Restricted cubic spline curve***

A restricted cubic spline curve was assessed in a logistic regression model to evaluate the association between body mass index (BMI) and post-stroke clinical outcomes. Knots of BMI were set at 18.5, 23, 25, and 30 kg/m^2^ according to the criteria of BMI for Asians proposed by the Regional Office for the Western Pacific Region of the World Health Organization. The cut-off value of BMI between underweight and normal weight was regarded as a reference. Patients with a BMI ≤11 kg/m^2^ and a BMI ≥35 kg/m^2^ were grouped into patients with a BMI of 11 kg/m^2^ and a BMI of 35 kg/m^2^, respectively.

***Decision tree ensemble model***

We used the R package xgboost (extreme gradient boosting [XGBoost] ver. 1.4.1.1; https://github.com/dmlc/xgboost) to construct a gradient boosting decision tree. In the gradient boosting decision tree, we tuned “nrounds” using 10-fold cross-validation and stopped the training with no improvement in the area under the receiver operating characteristic curve in test data after 5 rounds in a row. The other parameters were fixed as follows: “depth”, 6; “eta”, 0.1; “gamma”, 0; “colsample_bytree”, 1; “min_child_weight”, 1; and “subsample”, 1. To use the interpretability method SHapley Additive exPlanation (SHAP), we applied the R package xgboost. We developed the decision tree ensemble model using comprehensive data, including demographics (age and men), risk factors (hypertension, dyslipidemia, atrial fibrillation, diabetes mellitus, smoking, and drinking), comorbid conditions (kidney disease on dialysis, end-stage renal failure, allergy, arteriosclerosis obliterans, valvular disease, ischemic heart disease, cardiomyopathy, and dementia), a previous history (stroke and other cardiovascular diseases), a family history of stroke, the pre-admission mRS score, waist circumference, the onset-to-admission time, ambulance use, pre-stroke medication (anticoagulants, antiplatelets, antidyslipidemic drugs, antihypertensives, and antidiabetic drugs), physiological data (systolic blood pressure, diastolic blood pressure, and heart rate), brain imaging (diffusion-weighted imaging findings and site of lesions), stroke etiology, the NIHSS score on admission, laboratory data (white blood cell count, red blood cell count, hematocrit, hemoglobin, platelet count, and concentrations of aspartate aminotransferase, alanine aminotransferase, lactate dehydrogenase, alkaline phosphatase, total bilirubin, low-density lipoprotein, cholesterol, high-density lipoprotein cholesterol, triglycerides, total protein, creatine phosphokinase, blood urea nitrogen, creatinine, glucose, hemoglobin A1c, sodium, potassium, and high-sensitivity C-reactive protein, the estimated glomerular filtration rate, the international normalized ratio of prothrombin time, the activated partial thromboplastin time, fibrinogen concentrations, and D-dimer concentrations), and reperfusion therapy (intravenous recombinant tissue-type plasminogen activator and endovascular thrombectomy). The areas under the curves in predicting clinical outcomes post-stroke using the gradient boosting decision tree model were evaluated by the bootstrap method. The mean (standard deviation) areas under the curve were 0.855 (0.001) for a poor functional outcome, 0.865 (0.002) for functional disability, and 0.891 (0.005) for death at discharge. Additionally, the mean areas under the curve were 0.874 (0.001) for a poor functional outcome, 0.875 (0.001) for functional disability, and 0.838 (0.012) for death at 3 months. All statistical analyses were performed by using R statistical package (http://www.r-project.org/, version 4.1.0).

**References**

1 Seino, Y. *et al.* Report of the committee on the classification and diagnostic criteria of diabetes mellitus. *J Diabetes Investig* **1**, 212-228, (2010).

2 Adams, H. P., Jr. *et al.* Classification of subtype of acute ischemic stroke. Definitions for use in a multicenter clinical trial. TOAST. Trial of Org 10172 in Acute Stroke Treatment. *Stroke* **24**, 35-41, (1993).

**Table S1. SHAP values of BMI for unfavorable clinical outcomes at 3 months**

|  | Median (IQR) |
| --- | --- |
| Poor functional outcome |  |
| Underweight (11.8–18.4 kg/m^2^), n=916 | 0.18 (0.13–0.25) |
| Normal weight (18.5–22.9 kg/m^2^), n=4840 | -0.02 (-0.04–0.05) |
| Overweight (23.0–24.9 kg/m^2^), n=2673 | -0.04 (-0.06 to -0.04) |
| Obesity (25.0–60.5 kg/m^2^), n=3320 | -0.03 (-0.04 to -0.02) |
| Functional disability |  |
| Underweight (11.8–18.4 kg/m^2^), n=857 | 0.27 (0.21–0.38) |
| Normal weight (18.5–22.9 kg/m^2^), n=4728 | -0.01 (-0.05–0.07) |
| Overweight (23.0–24.9 kg/m^2^), n=2641 | -0.08 (-0.10 to -0.06) |
| Obesity (25.0–60.5 kg/m^2^), n=3285 | -0.05 (-0.08 to -0.02) |
| Death |  |
| Underweight (11.8–18.4 kg/m^2^), n=916 | 0.15 (0.11–0.22) |
| Normal weight (18.5–22.9 kg/m^2^), n=4840 | 0.00 (-0.01–0.02) |
| Overweight (23.0–24.9 kg/m^2^), n=2673 | -0.03 (-0.05 to -0.02) |
| Obesity (25.0–60.5 kg/m^2^), n=3320 | -0.03 (-0.05 to -0.02) |

SHAP values were calculated for each BMI category in the decision tree ensemble model for predicting unfavorable outcomes at 3 months.

SHAP: SHapley Additive exPlanation, BMI: body mass index, IQR: interquartile range.

**Table S2. Baseline characteristics according to BMI for analysis of discharge outcomes**

|  | Underweight  (<18.5 kg/m^2^)  n=939 | Normal weight  (18.5–22.9 kg/m^2^)  n=4934 | Overweight  (23.0–24.9 kg/m^2^)  n=2730 | Obesity  (≥25.0 kg/m^2^)  n=3386 | P | P_trend_ |
| --- | --- | --- | --- | --- | --- | --- |
| Age, y, mean±SD | 76±13 | 72±12 | 70±11 | 67±12 | <0.001 | <0.001 |
| Men, n (%) | 424 (45.2) | 3040 (61.6) | 1918 (70.3) | 2276 (67.2) | <0.001 | <0.001 |
| Risk factors |  |  |  |  |  |  |
| Hypertension, n (%) | 656 (69.9) | 3703 (75.1) | 2275 (83.3) | 2960 (87.4) | <0.001 | <0.001 |
| Diabetes, n (%) | 169 (18.0) | 1349 (27.3) | 835 (30.6) | 1313 (38.8) | <0.001 | <0.001 |
| Dyslipidemia, n (%) | 336 (35.8) | 2519 (51.1) | 1710 (62.6) | 2324 (68.6) | <0.001 | <0.001 |
| Atrial fibrillation, n (%) | 312 (33.2) | 1148 (23.3) | 534 (19.6) | 609 (18.0) | <0.001 | <0.001 |
| Waist circumference, cm, mean±SD | 69.6±6.3 | 79.3±6.4 | 86.0±5.8 | 94.2±8.7 | <0.001 | <0.001 |
| Previous stroke, n (%) | 143 (15.2) | 754 (15.3) | 431 (15.8) | 525 (15.5) | 0.94 | 0.70 |
| Pre-stroke modified Rankin Scale score of 1, n (%) | 178 (19.0) | 639 (13.0) | 290 (10.6) | 353 (10.4) | <0.001 | <0.001 |
| Stroke subtype, n (%) |  |  |  |  |  |  |
| Cardioembolism | 291 (31.0) | 1033 (20.9) | 465 (17.0) | 495 (14.6) | <0.001 | <0.001 |
| Small-vessel occlusion | 226 (24.1) | 1396 (28.3) | 840 (30.8) | 1116 (33.0) | <0.001 | <0.001 |
| Large artery atherosclerosis | 108 (11.5) | 790 (16.0) | 442 (16.2) | 580 (17.1) | 0.001 | 0.002 |
| Unclassified | 314 (33.4) | 1715 (34.8) | 983 (36.0) | 1195 (35.3) | 0.49 | 0.28 |
| Baseline NIHSS score, median (IQR) | 4 (1–9) | 2 (1–5) | 2 (1–4) | 2 (1–4) | <0.001 | <0.001 |
| Reperfusion therapy, n (%) | 131 (14.0) | 557 (11.3) | 282 (10.3) | 306 (9.0) | <0.001 | <0.001 |

BMI: body mass index, P_trend_: P for trend, SD: standard deviation, NIHSS: National Institutes of Health Stroke Scale, IQR: interquartile range.

**Table S3. Baseline characteristics according to BMI based on World Health Organization criteria for analysis of 3-month outcomes**

|  | Underweight  (<18.5 kg/m^2^)  n=916 | Normal weight  (18.5–24.9 kg/m^2^)  n=7513 | Overweight  (25.0–29.9 kg/m^2^)  n=2820 | Obesity  (≥30.0 kg/m^2^)  n=500 | P | P_trend_ |
| --- | --- | --- | --- | --- | --- | --- |
| Age, y, mean±SD | 76±13 | 71±12 | 68±12 | 61±14 | <0.001 | <0.001 |
| Men, n (%) | 415 (45.3) | 4862 (64.7) | 1925 (68.3) | 305 (61.0) | <0.001 | <0.001 |
| Risk factors |  |  |  |  |  |  |
| Hypertension, n (%) | 640 (69.9) | 5866 (78.1) | 2450 (86.9) | 452 (90.4) | <0.001 | <0.001 |
| Diabetes, n (%) | 162 (17.7) | 2146 (28.6) | 1059 (37.6) | 230 (46.0) | <0.001 | <0.001 |
| Dyslipidemia, n (%) | 329 (35.9) | 4156 (55.3) | 1900 (67.4) | 373 (74.6) | <0.001 | <0.001 |
| Atrial fibrillation, n (%) | 306 (33.4) | 1653 (22.0) | 521 (18.5) | 72 (14.4) | <0.001 | <0.001 |
| Waist circumference, cm, mean±SD | 69.6±6.3 | 81.7±7.0 | 92.3±6.7 | 104.8±10.3 | <0.001 | <0.001 |
| Previous stroke, n (%) | 137 (15.0) | 1161 (15.5) | 447 (15.9) | 61 (12.2) | 0.21 | 0.57 |
| Pre-stroke modified Rankin Scale score of 1, n (%) | 175 (19.1) | 908 (12.1) | 299 (10.6) | 45 (9.0) | <0.001 | <0.001 |
| Stroke subtype, n (%) |  |  |  |  |  |  |
| Cardioembolism | 284 (31.0) | 1473 (19.6) | 420 (14.9) | 59 (11.8) | <0.001 | <0.001 |
| Small-vessel occlusion | 215 (23.5) | 2175 (28.9) | 910 (32.3) | 184 (36.8) | <0.001 | <0.001 |
| Large artery atherosclerosis | 107 (11.7) | 1205 (16.0) | 485 (17.2) | 83 (16.6) | 0.001 | 0.002 |
| Unclassified | 310 (33.8) | 2660 (35.4) | 1005 (35.6) | 174 (34.8) | 0.78 | 0.61 |
| Baseline NIHSS score, median (IQR) | 4 (1–9) | 2 (1–5) | 2 (1–4) | 2 (1–3) | <0.001 | <0.001 |
| Reperfusion therapy, n (%) | 125 (13.6) | 820 (10.9) | 250 (8.9) | 45 (9.0) | <0.001 | <0.001 |

BMI: body mass index, P_trend_: P for trend, SD: standard deviation, NIHSS: National Institutes of Health Stroke Scale, IQR: interquartile range.

**Table S4. Associations between BMI based on World Health Organization criteria and unfavorable clinical outcomes at 3 months**

|  |  | Age- and sex-adjusted | | |  | Multivariable-adjusted | | |
| --- | --- | --- | --- | --- | --- | --- | --- | --- |
|  | Events, n (%) | OR | (95% CI) | P |  | OR | (95% CI) | P |
| Poor functional outcome |  |  |  |  |  |  |  |  |
| Underweight (11.8–18.4 kg/m^2^), n=916 | 375 (40.9) | 1.89 | (1.62–2.20) | <0.001 |  | 1.51 | (1.23–1.84) | <0.001 |
| Normal weight (18.5–24.9 kg/m^2^), n=7513 | 1631 (21.7) | 1.00 | Reference |  |  | 1.00 | Reference |  |
| Overweight (25.0–29.9 kg/m^2^), n=2820 | 501 (17.8) | 0.95 | (0.85–1.07) | 0.42 |  | 1.06 | (0.91–1.24) | 0.48 |
| Obesity (30.0–60.5 kg/m^2^), n=500 | 77 (15.4) | 1.08 | (0.83–1.40) | 0.58 |  | 1.43 | (1.01–2.02) | 0.04 |
| Functional disability |  |  |  |  |  |  |  |  |
| Underweight (11.8–18.4 kg/m^2^), n=857 | 316 (36.9) | 1.73 | (1.48–2.03) | <0.001 |  | 1.40 | (1.14–1.73) | 0.002 |
| Normal weight (18.5–24.9 kg/m^2^), n=7369 | 1487 (20.2) | 1.00 | Reference |  |  | 1.00 | Reference |  |
| Overweight (25.0–29.9 kg/m^2^), n=2790 | 471 (16.9) | 0.98 | (0.87–1.11) | 0.76 |  | 1.06 | (0.90–1.24) | 0.49 |
| Obesity (30.0–60.5 kg/m^2^), n=495 | 72 (14.5) | 1.10 | (0.84–1.45) | 0.47 |  | 1.39 | (0.98–1.99) | 0.07 |
| Death |  |  |  |  |  |  |  |  |
| Underweight (11.8–18.4 kg/m^2^), n=916 | 59 (6.4) | 2.87 | (2.08–3.95) | <0.001 |  | 2.01 | (1.34–3.00) | 0.001 |
| Normal weight (18.5–24.9 kg/m^2^), n=7513 | 144 (1.9) | 1.00 | Reference |  |  | 1.00 | Reference |  |
| Overweight (25.0–29.9 kg/m^2^), n=2820 | 30 (1.1) | 0.66 | (0.44–0.98) | 0.04 |  | 0.79 | (0.50–1.25) | 0.31 |
| Obesity (30.0–60.5 kg/m^2^), n=500 | 5 (1.0) | 0.83 | (0.34–2.06) | 0.69 |  | 1.11 | (0.40–3.08) | 0.85 |

ORs and 95% CIs of each outcome of interest were estimated for underweight, overweight, and obesity vs. normal weight as a reference. The multivariable model included age, sex, hypertension, diabetes mellitus, dyslipidemia, atrial fibrillation, waist circumference, prior stroke, pre-stroke functional status, stroke subtype, the National Institutes of Health Stroke Scale score on admission, and reperfusion therapy.

BMI: body mass index, OR: odds ratio, CI: confidence interval.

**Figure S1. Association between body mass index and a poor functional outcome at 3 months according to age, diabetes mellitus, and neurological severity**

ORs and 95% CIs of a poor functional outcome at 3 months are shown for underweight, overweight, and obesity in reference to normal weight according to age (<65 years and ≥65 years, upper panel), diabetes mellitus (middle panel), and neurological severity (NIHSS score on admission ≤6 and >6, lower panel). The multivariable model included age, sex, hypertension, diabetes mellitus, dyslipidemia, atrial fibrillation, waist circumference, prior stroke, pre-stroke functional status, stroke subtype, the NIHSS score on admission, and reperfusion therapy. P for heterogeneity was evaluated by adding an interaction term of the body mass index categories and each subgroup.

OR: odds ratio, CI: confidence interval, Ph: P for heterogeneity, NIHSS: National Institutes of Health Stroke Scale.

**Figure S2. SHAP values of body mass index for a poor functional outcome at 3 months according to age, diabetes mellitus, and neurological severity**

SHAP values of body mass index for a poor functional outcome at 3 months in individual patients are plotted according to age (<65 years and ≥65 years, left upper panel), diabetes mellitus (left middle panel), or neurological severity (NIHSS score on admission ≤6 and >6, left lower panel). Median SHAP values are also plotted vs. mean values of body mass index in each group (underweight, normal weight, overweight, and obesity) according to age (right upper panel), diabetes mellitus (right middle panel), or neurological severity (right lower panel). The size of the symbols represents the number of patients in each body mass index group.

SHAP: SHapley Additive exPlanation, NIHSS: National Institutes of Health Stroke Scale.

**Figure S3. Relationships between body mass index and unfavorable clinical outcomes at discharge**

The associations between body mass index and the risk of a poor functional outcome (A), functional disability (B), or death (C) at discharge are shown. ORs (solid lines) and 95% CIs (dotted lines) were estimated in reference to a body mass index of 18.5 kg/m^2^ using a logistic regression model with restricted cubic spline curves. The multivariable model included age, sex, hypertension, diabetes mellitus, dyslipidemia, atrial fibrillation, waist circumference, prior stroke, pre-stroke functional status, stroke subtype, the National Institutes of Health Stroke Scale score on admission, and reperfusion therapy.

OR: odds ratio, CI: confidence interval.

**Figure S4. SHAP values of body mass index for unfavorable clinical outcomes at discharge**

SHAP values of body mass index for predicting a poor functional outcome (A), functional disability (B), or death (C) at discharge in 11 body mass index categories are shown. SHAP values for post-stroke clinical outcomes were calculated in a decision tree ensemble model. Boxes, horizontal lines, and vertical lines indicate the interquartile range, median, and 5–95 percentiles, respectively, of SHAP values for each clinical outcome in the 11 groups according to body mass index in increments of 2 (body mass index groups: <16 kg/m^2^, increment of 2 from 16 to 33.9 kg/m^2^, and ≥34 kg/m^2^).

SHAP: SHapley Additive exPlanation.

**Figure S5. Flow chart of the patients**

ADL: activities of daily living, mRS: modified Rankin Scale.

**Appendix**

Steering Committee and Research Working Group Members: Takao Ishitsuka, MD, PhD (Fukuoka Mirai Hospital, Fukuoka, Japan), Setsuro Ibayashi, MD, PhD (Chair, Seiai Rehabilitation Hospital, Onojo, Japan), Kenji Kusuda, MD, PhD (Seiai Rehabilitation Hospital, Onojo, Japan), Kenichiro Fujii, MD, PhD (Japan Seafarers Relief Association Moji Ekisaikai Hospital, Kitakyushu, Japan), Tetsuhiko Nagao, MD, PhD (Safety Monitoring Committee, Seiai Rehabilitation Hospital, Onojo, Japan), Yasushi Okada, MD, PhD (Vice-Chair, National Hospital Organization Kyushu Medical Center, Fukuoka, Japan), Masahiro Yasaka, MD, PhD (National Hospital Organization Kyushu Medical Center, Fukuoka, Japan), Hiroaki Ooboshi, MD, PhD (Fukuoka Dental College Medical and Dental Hospital, Fukuoka, Japan), Takanari Kitazono, MD, PhD (Principal Investigator, Kyushu University, Fukuoka, Japan), Katsumi Irie, MD, PhD (Hakujyuji Hospital, Fukuoka, Japan), Tsuyoshi Omae, MD, PhD (Imazu Red Cross Hospital, Fukuoka, Japan), Kazunori Toyoda, MD, PhD (National Cerebral and Cardiovascular Center, Suita, Japan), Hiroshi Nakane, MD, PhD (National Hospital Organization Fukuoka–Higashi Medical Center, Koga, Japan), Masahiro Kamouchi, MD, PhD (Kyushu University, Fukuoka, Japan), Hiroshi Sugimori, MD, PhD (National Hospital Organization Kyushu Medical Center, Fukuoka, Japan), Shuji Arakawa, MD, PhD (Steel Memorial Yawata Hospital, Kitakyushu, Japan), Kenji Fukuda, MD, PhD (St Mary’s Hospital, Kurume, Japan), Tetsuro Ago, MD, PhD (Kyushu University, Fukuoka, Japan), Jiro Kitayama, MD, PhD (Fukuoka Red Cross Hospital, Fukuoka, Japan), Shigeru Fujimoto, MD, PhD (Jichi Medical University, Shimotsuke, Japan), Shoji Arihiro, MD (Japan Labor Health and Welfare Organization Kyushu Rosai Hospital, Kitakyushu, Japan), Junya Kuroda, MD, PhD (National Hospital Organization Fukuoka–Higashi Medical Center, Koga, Japan), Yoshinobu Wakisaka, MD, PhD (Kyushu University Hospital, Fukuoka, Japan), Yoshihisa Fukushima, MD (St Mary’s Hospital, Kurume, Japan), Ryu Matsuo, MD, PhD (Secretariat, Kyushu University, Fukuoka, Japan), Fumi Irie, MD, PhD (Kyushu University, Fukuoka, Japan), Kuniyuki Nakamura, MD, PhD (Kyushu University Hospital, Fukuoka, Japan), and Takuya Kiyohara, MD, PhD (Kyushu University Hospital, Fukuoka, Japan).
